# Run-off impacts on Arctic kelp holobionts have strong implications on ecosystem functioning and bioeconomy

**DOI:** 10.1038/s41598-024-82287-w

**Published:** 2024-12-16

**Authors:** Sarina Niedzwiedz, Claudia Schmidt, Yunlan Yang, Bertille Burgunter-Delamare, Sebastian Andersen, Lars Hildebrandt, Daniel Pröfrock, Helmuth Thomas, Rui Zhang, Børge Damsgård, Kai Bischof

**Affiliations:** 1 Marine Botany, Faculty of Biology and Chemistry & MARUM, University of Bremen, 28359 Bremen, Germany; 2 Institute for Chemistry and Biology of the Marine Environment (ICBM), University of Oldenburg, 26111 Oldenburg, Germany; 3 Department of Marine Carbon Cycles, Institute of Carbon Cycles, Helmholtz-Zentrum Hereon, 21502 Geesthacht, Germany; 4 Archaeal Biology Center, Synthetic Biology Research Center, Shenzhen Key Laboratory of Marine Microbiome Engineering, Key Laboratory of Marine Microbiome Engineering of Guangdong Higher Education Institutes, Institute for Advanced Study, Shenzhen University, Shenzhen, 518052 China; 5 Matthias Schleiden Institute of Genetics, Bioinformatics and Molecular Botany, Friedrich Schiller University Jena, 07743 Jena, Germany; 6 The University Centre of Svalbard (UNIS), Longyearbyen, 9171 Norway; 7 Department Inorganic Environmental Chemistry, Institute of Coastal Environmental Chemistry, Helmholtz-Zentrum Hereon, 21502 Geesthacht, Germany

**Keywords:** Biochemistry, Heavy metals, Holobiont, Microbial community, Rare earth elements, *Saccharina Latissima*, Ecology, Microbiology, Physiology, Climate sciences, Ecology

## Abstract

Kelps (Laminariales, Phaeophyceae) are foundation species along Arctic rocky shores, providing the basis for complex ecosystems and supporting a high secondary production. Due to ongoing climate change glacial and terrestrial run-off are currently accelerating, drastically changing physical and chemical water column parameters, e.g., water transparency for photosynthetically active radiation or dissolved concentrations of (harmful) elements. We investigated the performance and functioning of Arctic kelp holobionts in response to run-off gradients, with a focus on the effect of altered element concentrations in the water column. We found that the kelp *Saccharina latissima* accumulates harmful elements (e.g., cadmium, mercury) originating from coastal run-off. As kelps are at the basis of the food web, this might lead to biomagnification, with potential consequences for high-latitude kelp maricultures. In contrast, the high biosorption potential of kelps might be advantageous in monitoring environmental pollution or potentially extracting dissolved rare earth elements. Further, we found that the relative abundances of several kelp-associated microbial taxa significantly responded to increasing run-off influence, changing the kelps functioning in the ecosystem, e.g., the holobionts nutritional value and elemental cycling. The responses of kelp holobionts to environmental changes imply cascading ecological and economic consequences for Arctic kelp ecosystems in future climate change scenarios.

**Supplementary Information:**

The online version contains supplementary material available at 10.1038/s41598-024-82287-w.

## Introduction

Kelps (Laminariales, Phaeophyceae) act as foundation species from temperate to polar rocky shores^1^, governing ecosystem functioning, resilience and biodiversity^2^. Kelps are important primary producers in coastal zones^2^, supporting high secondary production, e.g. microbes, invertebrates, fish and mammals^3^, which also makes them economically important^1,4^. As consequence of ongoing climate change, a shift in the biogeographical extent has been monitored, as well as a change in their productivity on local and global scales^5,6^.

In the Arctic, many factors driving kelp forest dynamics are not well understood^7^, even though climate change is especially pronounced. The rate of temperature rise is far beyond the global average rate^8–10^. Thereby, high Arctic coastlines have recently become habitable for cold-temperate kelps, such as *Saccharina latissima*^11–13^ and an overall future range expansion of kelps to higher latitudes is expected^14–16^. However, rising temperatures also cause an accelerating glacial melt, thawing permafrost and higher precipitation rates^17–19^, leading to higher discharge of freshwater and terrestrial material into Arctic fjords^20^. These run-off plumes alter physical water conditions. Temperature and salinity differences between the run-off and marine water masses stratify the fjord water column, establishing strong gradients^21^. Increased concentrations of suspended particles result in a darkening of Arctic fjords in summer^22–24^. Changes in physical water conditions affect benthic primary producers in the adjacent ecosystems, resulting in a shift in the kelp forest community and a significant shoaling of the kelp forest since 1996^25^.

Further, run-off alters the chemical properties of the water column, washing organic matter, and littoral material^26,27^, as well as a wide range of legacy pollutants into the fjord^28^. Consequently, increased concentrations of biologically harmful elements, such as dissolved mercury (*d*Hg) were also detected in freshwater discharge^29^. Kelps were shown to have a high biosorption potential for ions from seawater^30^. Consequently, they take up dissolved heavy metals^30,31^, either intracellularly during their growth phase^32^ or via cell wall incorporated alginates and fucoidan^31^. The heavy metal ion uptake and accumulation are highly dependent on temperature, pH, dissolved metal concentration in the water column, presence of competing metal ions and exposure time (reviewed by^32^). A high mass fraction of heavy metals (e.g., *b*Cu, *b*Pb) has a highly destructive potential by inducing oxidative stress, which was shown to decrease kelp performance, e.g., reduced growth rates^33^. The high biosorption potential of kelps for cations, also results in an accumulation of macro- and micronutrients, resulting in a high nutritional value of kelps^34^.

Kelps are a hotspot for microscopic biodiversity^35^. The kelp-associated microbial communities can serve as an indicator of kelp health, as they are largely dependent on host conditions, changing when the kelp is stressed^36,37^. Further, the microbial communities depend on environmental factors^38^. Heterotrophic bacteria provide a crucial link in the food web, connecting kelp primary production with kelp consumers, by degrading particulate organic matter that kelps release^39,40^. Hence, environmental changes affecting the kelp holobiont might have consequences for the entire food web and ecosystem.

**Fig. 1:**
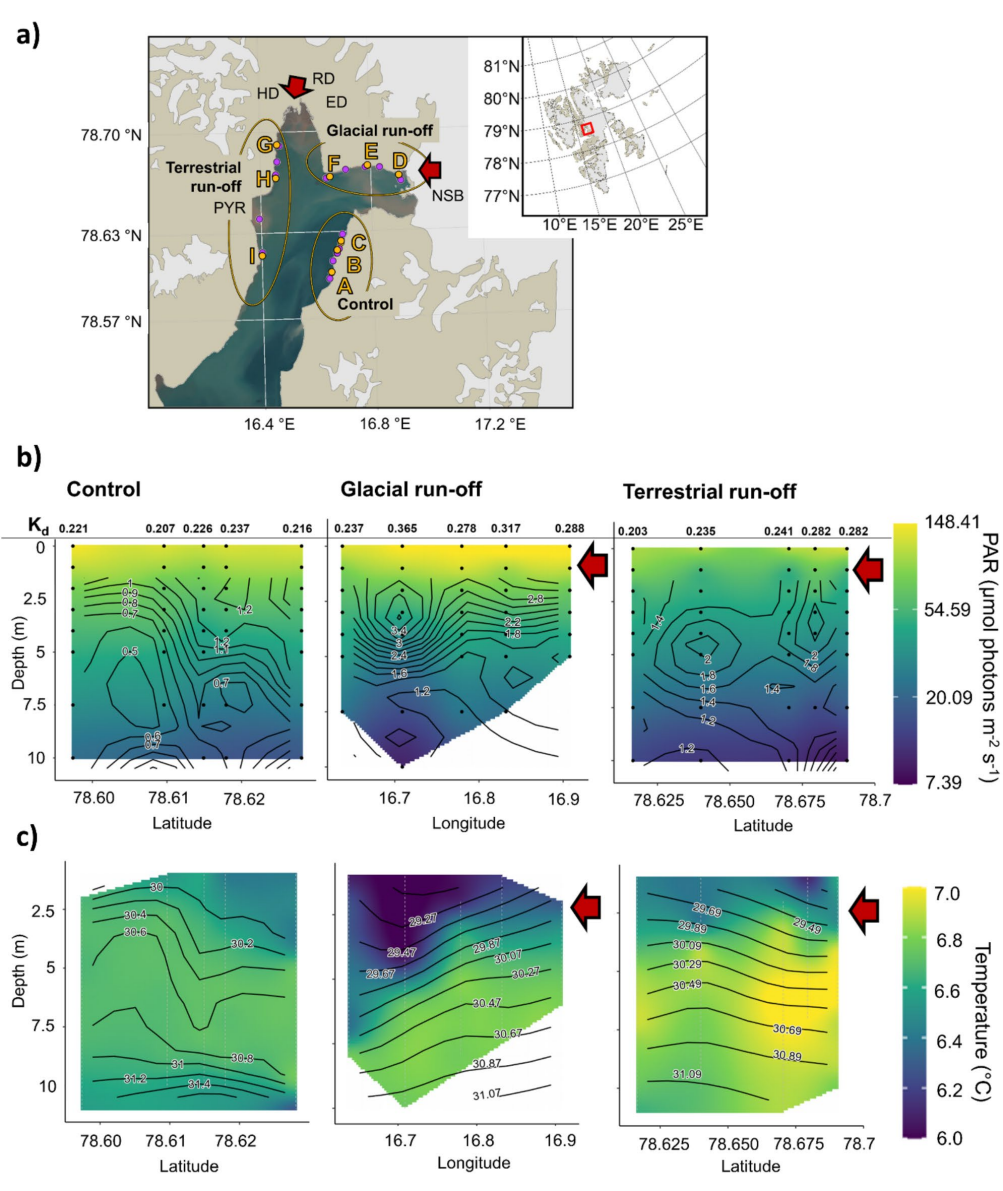
Environmental parameters at the study site in Billefjorden, Svalbard. (**a**) Map of Billefjorden, Svalbard. Right upper corner: overview map of Svalbard; red rectangle: Billefjorden. A–C: Control area. D–F: Glacial run-off area; NSB: Nordenskjölbreen. G–I: Terrestrial run-off area; ED: Ebbadalen; HD: Hørbyedalen; RD: Ragnardalen; PYR: Pyramiden (uninhabited miner’s settlement). Yellow points, A–I: Position of kelp sampling. Purple points: Positions of CTD/PAR measurements. Red arrows: direction of run-off inflow. Map: RStudio, PlotSvalbard^101^. Satellite image fjord water: toposvalbard.npolar.no; 06.02.2023. (**b**,**c**) Section plots of environmental conditions in sampling areas in Billefjorden (control, glacial run-off, terrestrial run-off) from 0–10 m depth on 30th August 2022. Red arrow: direction of run-off inflow to the run-off areas. Note the different x-axis. Black dots (**b**) / white vertical lines (**c**): actual PAR/CTD measurements in water column. White areas within section plots: insufficient data to support the model. (**b**) Colour gradient: photosynthetically available radiation (PAR; µmol photons m^−2^ s^−1^; scale as log to highlight low PAR intensities). Contour: turbidity (NTU). Numbers above depth transects: PAR attenuation coefficient (K_d_). **c**) Colour gradient: temperature (°C). Contour: salinity (S_A_).

The aim of this in-situ study is to assess the impact of run-off on Arctic kelp holobionts functioning to draw conclusions on future Arctic coastal ecosystems and potential bioeconomic impacts. We collected *S. latissima* sporophytes at the end of the run-off season in Billefjorden, Svalbard. We compared kelp specimens that were influenced by glacial run-off (samples were collected with increasing distance to a sea-terminating glacier) or terrestrial run-off (samples were collected with increasing distance to a land-terminating glacier) to a control area (samples from relatively clear coastal water) (Fig. 1a). We analysed the elemental composition of kelps, their biochemical response and associated microbial community and related these responses to physical and chemical water column mapping. The findings of our study have implications for present-day spatial variability as well as near future temporal changes. Our study was guided by three hypotheses: (I) A high concentration of dissolved elements in run-off results in higher elemental mass fractions in kelps. (II) As heavy metals, such as Cu and Pb, have been shown to lead to oxidative stress in algae^33^, we expect high heavy metal mass fractions to correlate with higher antioxidant activities. (III) The kelp-associated microbial community will be influenced by different environmental and host conditions in the respective areas, resulting in altered microbial species composition.

## Results

For overview reasons, statistical results of the analysis of variance are displayed in Table 1 and are therefore not given in the text. Kelp parameters were analysed in response to sampling area (control area, glacial run-off area, terrestrial run-off area) and sampling station (A–I) (Fig. 1a).

**Table 1:** Statistical results of kelp responses.

Parameter	Fixed effect	numDF	denDF	F value	*P* value
Biogenic elemental mass fraction					
Aluminium (Al)	Area	2	68	23.1	**< 0.001**
	Station	8	61	10.6	**< 0.001**
Iron (Fe)	Area	2	66	30.7	**< 0.001**
	Station	8	61	9.1	**< 0.001**
Manganese (Mn)	Area	2	67	20.6	**< 0.001**
	Station	8	61	20.7	**< 0.001**
Copper (Cu)	Area	2	69	13.2	**< 0.001**
	Station	8	63	5.8	**< 0.001**
Cobalt (Co)	Area	2	68	1.2	0.3
	Station	8	60	5.3	**< 0.001**
Cadmium (Cd)	Area	2	69	5.6	**0.006**
	Station	8	63	2.9	**0.007**
Lead (Pb)	Area	2	68	16.1	**< 0.001**
	Station	8	62	8.2	**< 0.001**
Mercury (Hg)	Area	2	68	53.1	**< 0.001**
	Station	8	61	28.8	**< 0.001**
Biochemistry					
Chlorophyll *a*	Area	2	69	6.5	**0.002**
	Station	8	62	3.1	**0.006**
De-epoxidation state of xanthophyll cycle pigments (DPS)	Area	2	68	14.5	**< 0.001**
	Station	8	63	5.1	**< 0.001**
Antioxidant activity	Area	2	69	8.5	**< 0.001**
	Station	8	63	3.8	**0.001**
Biodiversity indices					
Shannon entropy	Area	2	64	7.0	**0.002**
	Station	8	57	5.4	**< 0.001**
Pielou evenness	Area	2	63	15.5	**< 0.001**
	Station	8	57	6.2	**< 0.001**
Relative abundance of microbial taxa					
Bacteroidetes	Area	2	64	9.4	**< 0.001**
	Station	5	58	5.77	**0.001**
Proteobacteria	Area	2	64	4.4	**0.02**
	Station	8	55	9.5	**< 0.001**
Alphaproteobacteria	Area	2	64	0.07	0.93
	Station	8	56	4.82	**< 0.001**
Gammaproteobacteria	Area	2	64	4.55	**0.01**
	Station	8	58	5.05	**< 0.001**
Flavobacteria	Area	2	64	12.2	**< 0.001**
	Station	8	58	6.8	**< 0.001**
Tiotrichales	Area	2	62	18.2	**< 0.001**
	Station	8	56	4.42	**< 0.001**
Pirellulales	Area	2	61	17.3	**< 0.001**
	Station	8	55	5.74	**< 0.001**
Planctomycetes	Area	2	61	17.3	**< 0.001**
	Station	8	55	5.74	**< 0.001**
Saprospirales	Area	2	64	3.7	**0.03**
	Station	8	56	3.3	**0.003**
Rhodobacterales	Area	2	64	0.07	0.93
	Station	8	56	4.8	**< 0.001**

Results of analysis of variance (ANOVA). As the data met all requirements (normality, homoscedasticity), a linear model was fit on each response parameter. Sampling area and station (Fig. 1a) were modelled as single fixed effect to analyse spatial differences of kelp responses. Analysis of variance was tested on the model using the “anova” function (type I sums of squares). Significant results are marked in bold. Note: tested values are the means of replicates (Area: *N* = 3; Station: *N* = 6–9) numDF: numerator degrees of freedom. denDF: denominator degrees of freedom.

### Environmental parameters show run-off influence

Water column mapping revealed area-specific differences in the photosynthetically active radiation (PAR; µmol photons m^−2^ s^−1^), the PAR attenuation coefficient (K_d_) and turbidity (Fig. 1b), as well as in temperature (°C) and salinity (S_A_) (Fig. 1c). Turbidity was measured in Nephelometric Turbidity Units (NTU). The water column of the control area was not stratified. In the control area, we detected a comparably high PAR at 5 m water depth (42.2 ± 3.7 µmol photons m^−2^ s^−1^), hence low PAR attenuation (K_d_; 0.22 ± 0.01), as well as low turbidity values (0.5–1.6 NTU). Temperature ranges (6.3–6.7 °C) were low. Highest salinities were measured in the control area (S_A_ = 31.8). In both glacial and terrestrial run-off areas, PAR intensities at 5 m water depth were lower and K_d_ and turbidity higher than in the control area (Glacial run-off area: 35.4 ± 5.6 µmol photons m^−2^ s^−1^, 0.30 ± 0.05; 1.0–3.6 NTU; Terrestrial run-off area: 30.7 ± 2.8 µmol photons m^−2^ s^−1^, 0.25 ± 0.03; 0.9–3.0 NTU). Further, the water column was markedly stratified. Temperature and salinity values were lower towards the surface (~ 0–3 m) compared to 10 m water depth. Temperature ranges were highest in the glacier run-off area (6.0–6.8 °C). Warmest temperatures were measured in the terrestrial run-off area (7.1 °C). Salinity differences resembled temperature patterns, with lowest salinities close to the surface, increasing with increasing water depth. Lowest salinities were measured in the terrestrial run-off area (S_A_ = 28.9).

### Kelp elemental composition changes with run-off intensity

**Fig. 2:**
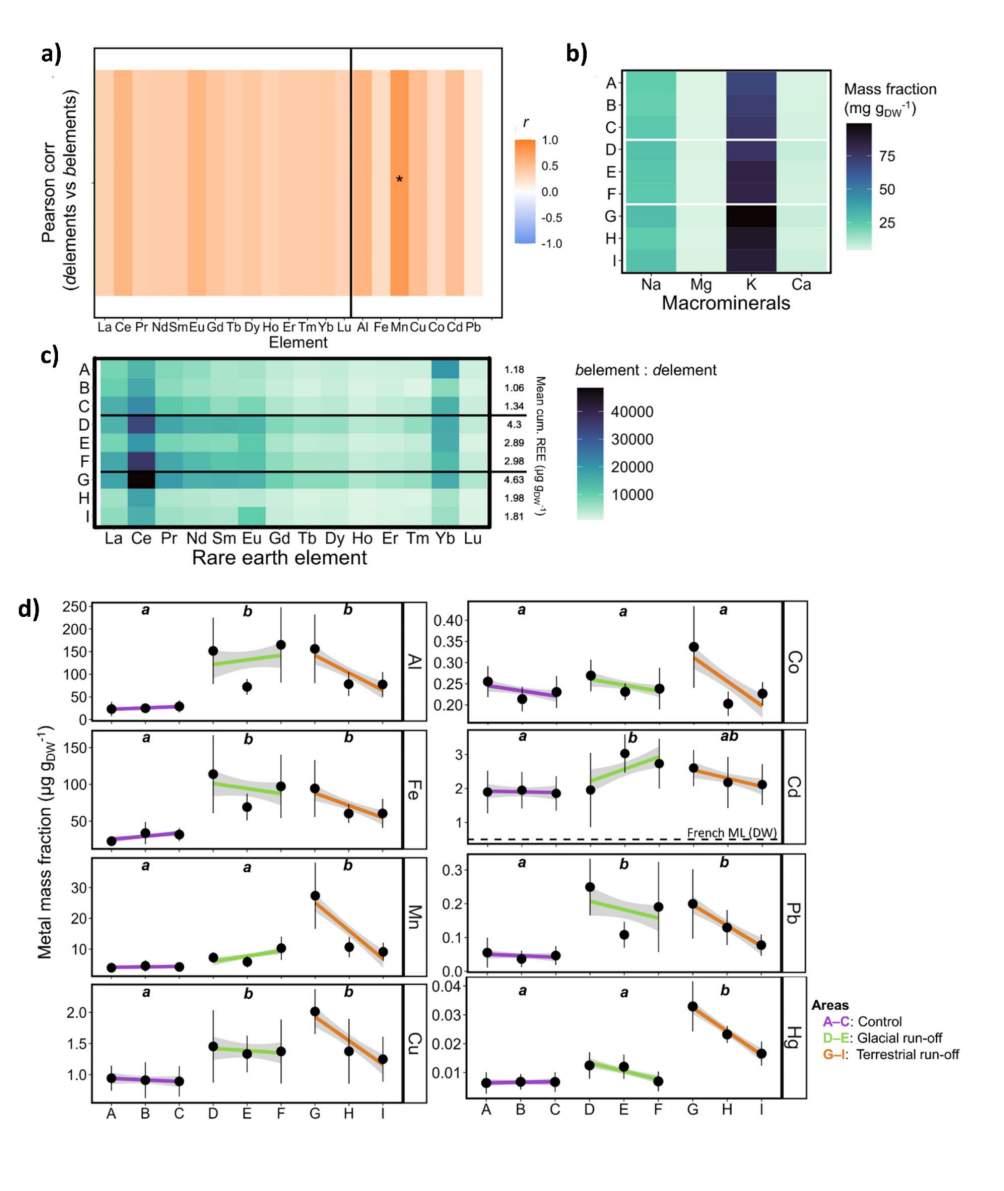
Element mass fraction in kelps. The elemental mass fraction was analysed in response to control (purple, ABC), glacial run-off (green, DEF) and terrestrial run-off area (orange, GHI) in Billefjorden (*N* = 6–9 per sampling station). DEF and GHI are ordered with increasing distance to run-off inflow (see Fig. 1A; distance between sampling stations A–I on x-axis are not to scale). (**a**) Pearson correlation coefficient *r* between dissolved element concentrations in the water column (5 m depth) and biogenic element mass fractions in kelps. Asterisk: Significant correlation (* *P* < 0.05). (**b**) *b*Element mass fraction of macrominerals (mg g_DW_^−1^) in kelps. (**c**) Ratio between biogenic (µg g_DW_^–1^) and dissolved (µg mL^−1^) rare earth elements (REE;^89^). Right side of the plot: mean cumulative REEs per sampling station (µg g_DW_^−1^). (**d**) *b*Metal mass fraction (µg g_DW_^−1^). Linear trend line: visualisation of elemental mass fraction (µg g_DW_^−1^) gradient in sampling area. Grey area: 95% confidence interval. Different letters within plots: significances between sampling area. Cd subplot: Maximum levels (ML) after Banach et al.^79^, based on French recommendations^106^.

Dissolved elements in the water column are marked with a prefixed *d* (Supplementary Fig. 1), while the biological elements in kelps are marked with a prefixed *b* (Supplementary Fig. 2). Dissolved element concentrations in 5 m water depth correlated positively with *b*element mass fraction in kelps. This correlation was significant for Mn (Fig. 2a). Overall sampling stations the macrominerals *b*K > *b*Na > *b*Ca > *b*Mg were the most abundant *b*elements with mass fractions between ~ 5–80 mg g_DW_^−1^ (Fig. 2b). Rare earth elements (*b*La–*b*Lu) showed higher mass fractions in kelps that were influenced by run-off inflow compared to the control area. The ratio between biological and dissolved rare earth elements ranged between 920 in Ho and 48,500 in Ce (Fig. 2c). The mean cumulative mass fraction of all rare earth elements ranged between 1.06 and 4.63 µg g_DW_^−1^.

The mass fractions of *b*Al, *b*Fe, *b*Mn, *b*Cu, *b*Co, *b*Cd, *b*Pb and *b*Hg (µg g_DW_^−1^) can be seen in Fig. 2d. Except for *b*Co, all element mass fractions were significantly affected by sampling area. For each area, distinct overall patterns were observed. In the control area, the mean mass fraction of all *b*elements was the lowest. Except for *b*Co, there are no significant mass fraction differences within the area, independent of the sampling station. For the glacial run-off area, the *b*elemental mass fraction of sampling station E was significantly lower compared to D or F for all elements except *b*Cd and *b*Hg. Except for *b*Al, *b*Mn and *b*Cd, there was a trend of decreasing element mass fraction with increasing distance to the meltwater run-off inflow. Compared to the other areas, mean element mass fractions of kelps from the terrestrial run-off area were highest, except for *b*Fe, *b*Cd and *b*Pb. Within the area, elemental mass fractions are significantly decreasing with increasing distance to the meltwater run-off inflow, except for *b*Mn and *b*Cd. The *b*Hg mass fraction in the terrestrial run-off area had the highest relative difference to the other areas, being ~ 60% higher compared to the glacial run-off area and ~ 72% higher compared to the control area.

### Kelp biochemical composition changes with run-off intensity

**Fig. 3:**
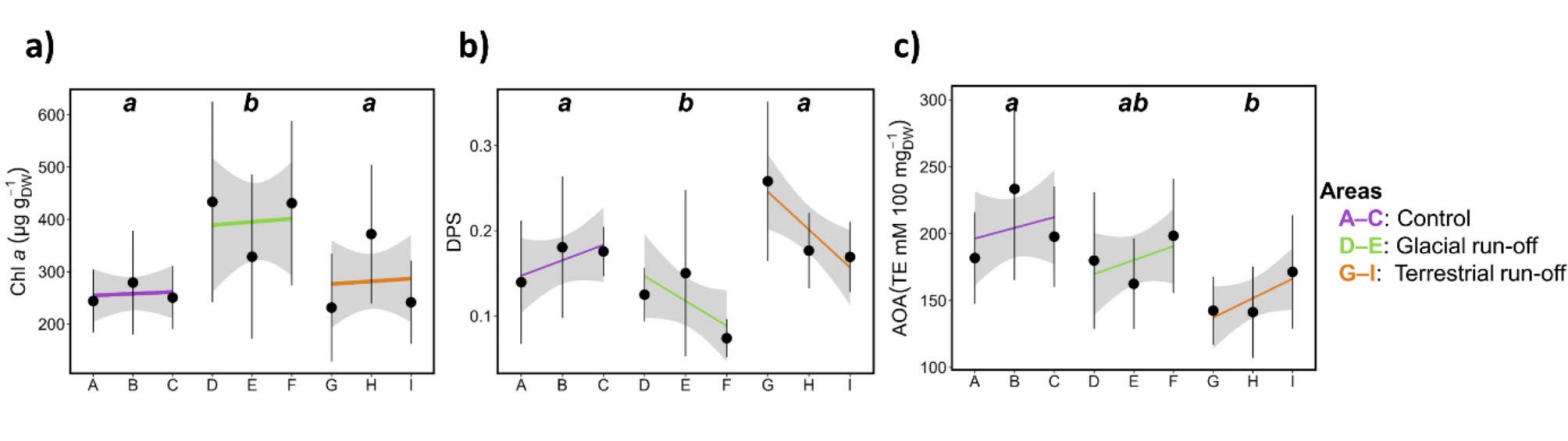
Biochemical response of kelps. The kelps biochemical response was analysed in response to control (purple, ABC), glacial run-off (green, DEF) and terrestrial run-off area (orange, GHI) in Billefjorden (*N* = 6–9 per sampling station). DEF and GHI are ordered with increasing distance to run-off inflow (see Fig. 1A; distance between sampling stations A–I on x-axis are not to scale). Different letters within plots: significances between each area. Linear trend line: visualisation of biochemical composition gradient in sampling area. Grey area: 95% confidence interval. (**a**) Chlorophyll *a* (µg g_DW_^−1^). (**b**) DPS: De-epoxidation state of xanthophyll cycle pigments. (**c**) AOA: Antioxidant activity (TE mM 100 mg_DW_^−1^).

The chlorophyll *a* content was significantly affected by sampling area and station (Fig. 3a). Mean kelp chlorophyll *a* of the sampling stations ranged between 232 and 431 µg g_DW_^−1^. The chlorophyll *a* content was significantly higher in the glacial run-off area (384 ± 173 µg g_DW_^−1^) compared to the control (258 ± 74 µg g_DW_^−1^) and terrestrial run-off area (282 ± 121 µg g_DW_^–1^). Within the areas, no overall pattern could be detected.

The de-epoxidation state of xanthophyll cycle pigments (DPS) varied significantly between all areas (Fig. 3b). DPS was highest in the terrestrial run-off area (0.20 ± 0.07) and lowest in the glacial run-off area (0.10 ± 0.04). Within the glacial run-off area, DPS significantly decreased with increasing distance to the run-off inflow. Within the terrestrial run-off area, this was seen as a trend.

The antioxidant activity (Fig. 3c) differed significantly between areas, being higher in the control area (204 ± 52 TE mM 100 mg_DW_^−1^) than in the glacial (181 ± 44 TE mM 100 mg_DW_^−1^) and the terrestrial run-off area (152 ± 36 TE mM 100 mg_DW_^−1^). We detected no significant differences within the run-off areas.

### Kelp-associated microbial community changes with run-off intensity

**Fig. 4:**
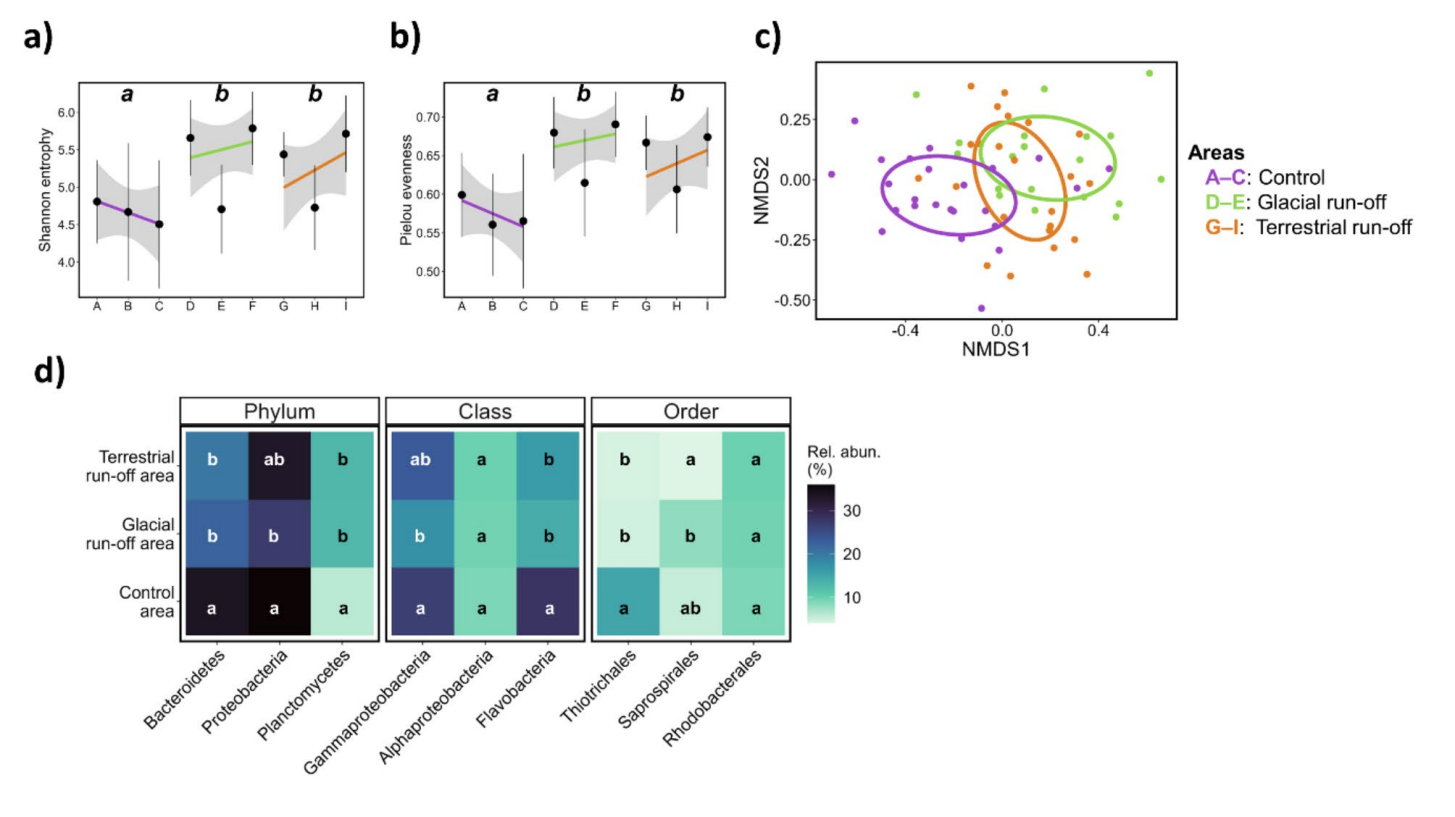
Kelp-associated microbial community. The microbial community was analysed in response to control (purple, ABC), glacial run-off (green, DEF) and terrestrial run-off area (orange, GHI) in Billefjorden (*N* = 6–9 per sampling station). DEF and GHI are ordered with increasing distance to run-off inflow (Fig. 1A; distance between sampling stations A–I on x-axis are not to scale). Different letters within plots: significances between sampling areas. (**a**,**b**) Linear trend line: visualisation of diversity indices in sampling areas. Grey area: 95% confidence interval. (**a**) Shannon diversity index. (**b**) Pielou evenness. (**c**) Non-metric multidimensional scaling (nMDS) ordinations showing the relative abundance of kelp-associated microbial groups in each area based on Bray-Curtis dissimilarities (stress value ≤ 0.05). (**d**) Heatmap showing the mean relative abundance of kelp-associated microbial community taxa (≥ 1%) in each sampling area.

Across all samples, we identified 4457 Amplicon Sequence Variants (ASVs). Both Shannon entropy and Pielou evenness were significantly affected by sampling area, being lower in the control area compared to the glacial and terrestrial run-off area (Fig. 4a, b).

Kelp-associated microbial communities showed distinct clustering between areas (Fig. 4c). All microbial taxa were significantly affected by sampling area and station (*P* < 0.05), except the class Alphaproteobacteria and the order Rhodobacterales. Most significant differences between sampling stations were across areas, hence, showing no clear patterns within areas. All kelp-associated microbial communities were dominated by Proteobacteria (especially Gammaproteobacteria) and Bacteriodetes (~ 58%; Fig. 4d). Their abundance was highest in the control area, compared to the glacial and terrestrial run-off area. The same pattern was observed for Flavobacteria (Control: 28.2 ± 12.6%; Glacial run-off: 14.4 ± 9.7%; Terrestrial run-off: 16.2 ± 7.8%) and Tiotrichales (Control: 15.1 ± 10.9%; Glacial run-off: 4.8 ± 4%; Terrestrial run-off: 3.1 ± 3.5%). Planctomycetes (Control: 4.4 ± 2.9%; Glacial run-off: 12.9 ± 4.9%; Terrestrial run-off: 10.7 ± 6.1%) had a lower abundance in the control area compared to the run-off dominated areas. The Saprospirae differed significantly between glacial (8.1 ± 6.7%) and terrestrial run-off areas (4.2 ± 4.1%), both not differing significantly from the control area (5.5 ± 3.3%). The Rhodobacterales showed no spatial variation in their abundance. The relative abundance of the free-living microbial community is displayed in Supplementary Fig. 3.

### High ecological variability between run-off systems

**Fig. 5:**
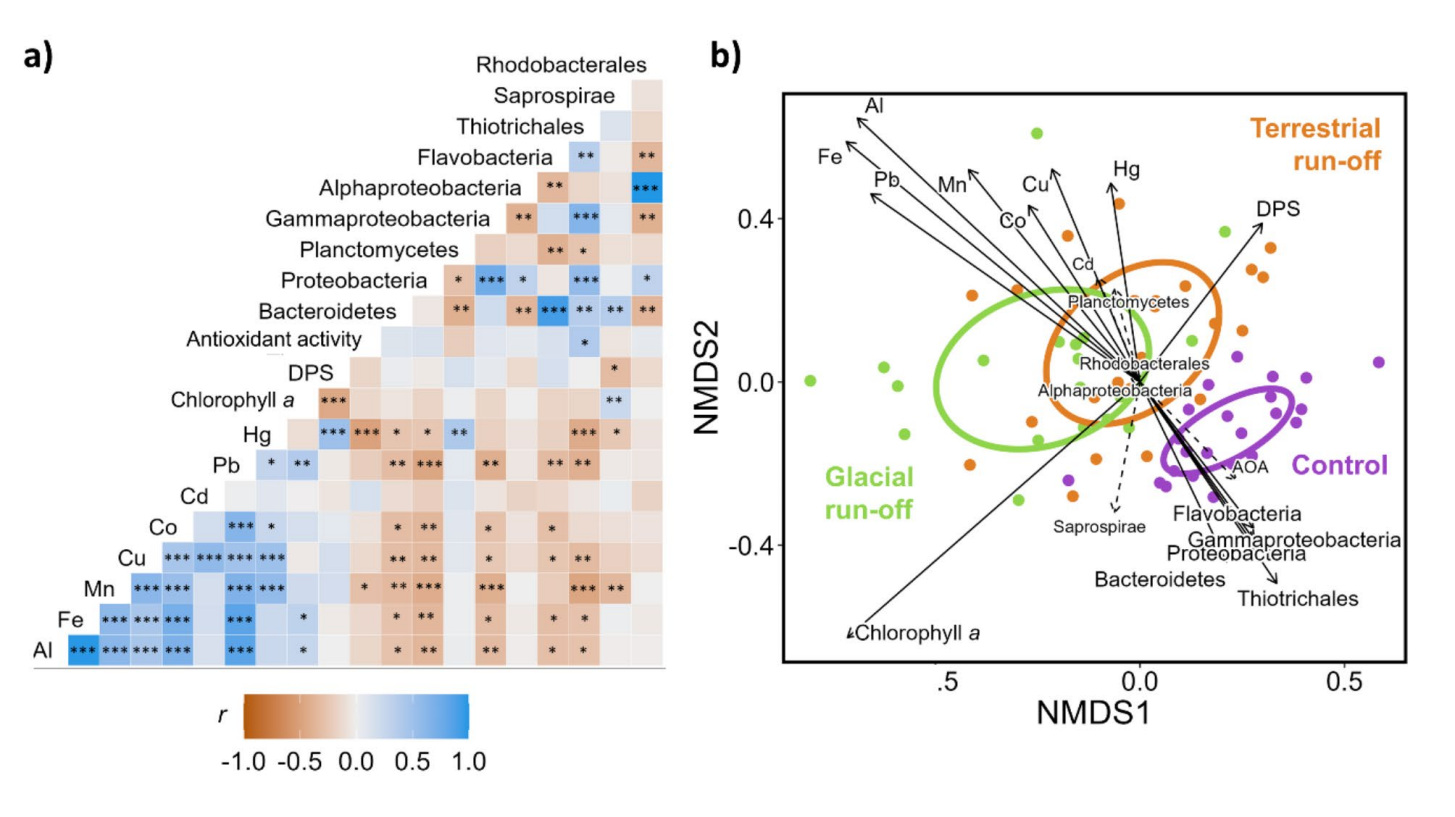
Correlations between response parameters. (**a**) Linear dependency between kelp response parameters. Colour scale: Pearson correlation co-efficient (*r*). Asterisk: significance of correlation (*: *P* < 0.05; **: *P* < 0.01; ***: *P* < 0.001. (**b**) Non-metric multidimensional scaling (nMDS) ordinations showing kelp responses based on Bray-Curtis dissimilarities. AOA: Antioxidant activity. Significant vectors: solid arrow, larger labelling (stress value ≤ 0.01). Non-significant vectors: dashed arrow, smaller labelling.

Kelps significantly differed in their elemental composition, biochemical response, and associated microbial community (Fig. 5). Their clustering coincided with spatial changes in run-off intensity and associated water parameters (e.g., temperature, PAR availability, dissolved element concentrations). Responses of kelps from glacial and terrestrial run-off areas were more similar to each other, than to the control area. Metal mass fractions in kelps correlated positively with each other and negatively with the relative abundance of microbial taxa, with many of the correlations being significant. The antioxidant activity correlated negatively with biogenic metal mass fraction in kelps, which was significant for Hg and Mn.

## Discussion

In the Arctic, global climate change causes glaciers and permafrost to melt, and increases precipitation rates^17–19^, resulting in extensive run-off plumes dominating many coastal areas^23^. Run-off plume differences relate both to present-day spatial variations, with the run-off plume influence being highest in the inner fjord region^21^; as well as near-future temporal changes, with glaciers retreating^41,42^. Run-off plumes were shown to change the water column physical parameters and carry nutrients, but also be the origin of harmful elements, such as heavy metals^26,43,44^. Along Arctic rocky coastlines, kelp holobionts function as foundation species, providing the basis for many associated species. If and how kelp holobiont health and functioning changes with variation in run-off is largely unknown, even though their responses can have cascading consequences for the entire ecosystem^39,40^.

**Fig. 6:**
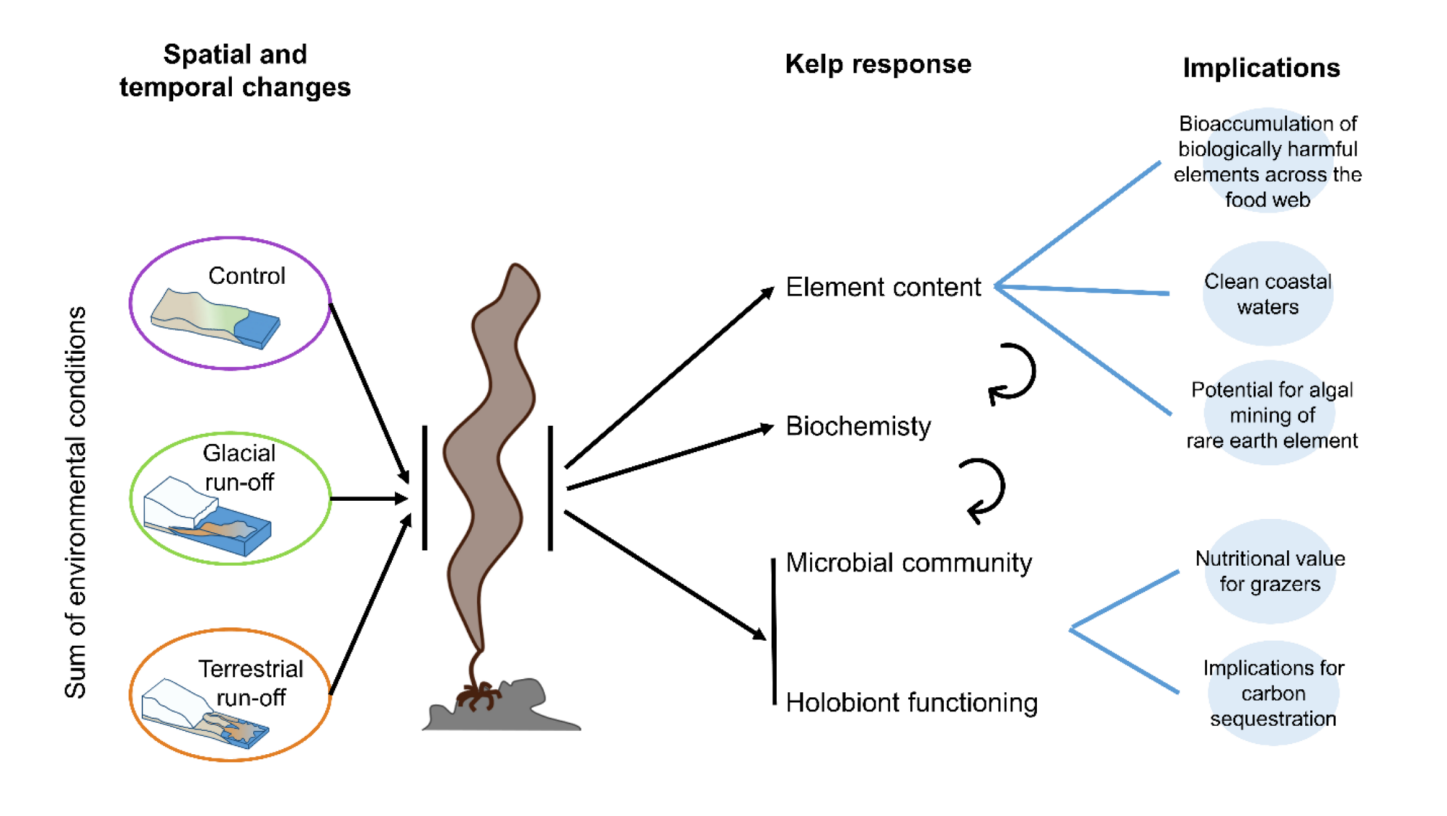
Major findings of the study, summarising the run-off influences on Arctic coastal ecosystem functioning with possible bioeconomic consequences. The target areas of this study (control area, glacial run-off, terrestrial run-off) relate to both present-day spatial differences and near-future temporal changes, when glaciers retreat. Responding to the sum of environmental conditions in each area (temperature, salinity, PAR availability, dissolved element concentration), we found significant changes in the kelp element content, biochemistry, and associated microbial community. While we cannot prove the causal dependency between the response parameters (but only correlations), their individual changes pose extensive consequences for high trophic levels, the ecosystem element cycling but also bioeconomic potential.

We found the element content, biochemistry and associated microbial community of *Saccharina latissima* to strongly respond to changes in run-off influence (clear water vs. glacial and terrestrial run-off), implying a high ecological variability between run-off systems (Figs. 5 and 6). In accordance with hypothesis I, we found the dissolved element concentration in the water column to correlate positively with element mass fractions in *S. latissima* specimens. Contradicting hypothesis II, the element mass fractions in kelps correlated negatively with the antioxidant activity. This might be due to the degradation of antioxidants due to chronic heavy metal exposure^45^. Corresponding to hypothesis III, we detected significant differences in the abundances of many kelp-associated microbial taxa between sampling areas, correlating negatively with *b*element mass fraction. Altered relative abundances of the microbial community relate to changes in the nutritional value of kelps and the ecosystems elemental cycling, e.g. in carbon.

In this interdisciplinary approach, we highlight the complexity of ecological interactions, presenting new connections and implications of run-off influence on Arctic coastal ecosystem functioning. In the following, we discuss the possible ecological consequences and present potential bio-economical perspectives of our findings. As run-off is predicted to accelerate in the future, the run-off induced changes in Arctic coastal ecosystems are likely to intensify.

By mapping the environmental conditions of the water column within each area, we validated run-off presence in the glacial and terrestrial run-off area, resulting in a stratified water column. In contrast, the water column in the control area was well-mixed. The generally small temperature and salinity variation in all areas (Fig. 1) can be attributed to the sampling campaign being conducted in late August, coinciding with the end of the run-off season^46^, with weak run-off plumes being present. However, the sampled kelps were exposed to the environmental conditions of their respective area during the entire run-off season. Hence, we argue that our chosen sampling areas are suitable to draw general conclusions about run-off effects on Arctic kelp holobiont functioning.

Being exposed to different environmental conditions such as PAR, temperature, salinity and dissolved element concentrations, we found that *S. latissima* strongly responded to the environmental conditions at the different sampling areas (Fig. 5b). This confirms the conclusions of Diehl et al.^47^, reviewing that *S. latissima* is highly plastic in its environmental response. Further, significant variations across all measured kelp responses imply a high ecological variability between run-off systems.

Seaweeds have long been known for their ability to accumulate ions from the surrounding water column^48^. We confirmed this by showing that the mass fraction of all elements in kelps correlated positively with dissolved elements in the water column (Fig. 2a), indicating that kelps accumulated elements from run-off discharge. However, this correlation was not significant for most elements. We attribute this to the high variability of run-off plumes and the dissolved element concentrations being only a point measurement of the whole run-off season. The accumulation of ions makes seaweeds good food sources for macrominerals, e.g. *b*Na, *b*Mg, *b*K and *b*Ca, which we detected to be the most abundant *b*elements in kelps^31,49^ (Fig. 2b).

In addition to macrominerals, we found higher metal mass fractions with higher run-off influence, such as *b*Al, *b*Fe, *b*Mn, *b*Cu, *b*Co (Fig. 2d). Many metals have physiological roles in algae, e.g. stabilising protein structures, catalying enzymatic reactions or facilitating electron transport^50^. Twining and Baines^51^ review the requirements of trace metals for marine phytoplankton, e.g. describing *b*Fe and *b*Mn to be essential in the photosynthetic electron transport chain. Overall, the concentration of these essential (trace) metals in the ocean is low and can even limit primary production^52^. As Arctic run-off plumes are characterised by increased concentrations of trace metals^27,29^, these habitats might support algal growth. However, we also detected harmful elements, such as *b*Cd and *b*Pb in the run-off influenced kelps. Thereby, the toxicity of metals seems to be related to the production of reactive oxygen species and an unbalanced cellular redox status^53^. Generally, seaweeds are able to accumulate a certain amount of heavy metals without any toxic effect, as their polysaccharides chelate them^48^. Nevertheless, negative effects of heavy metals on their morphology, growth, or photosynthetic and metabolic processes have been described. Cd, Pb or Hg can substitute essential trace elements in proteins or enzymes^54^, and the present study found them in significantly higher mass fractions in the run-off influenced kelps compared to the control area. In freshwater green algae, Al was described to lead to chloroses, necrosis and tissue weakening^55^. The interactive stress of Cu and Cd treatments resulted in overall reduced growth in *Macrocystis pyrifera*^56^. Kumar et al.^57^ described cascading antioxidant responses to mitigate Cd toxicity in the green alga *Ulva lactuca*. Ahamad & Shuhanija^58^ reported severe reductions in the maximum quantum yield of photosystem II and membrane disruption as responses of a red alga to an increased Hg mass fraction. Costa et al.^33^ classified Cu and Pb as stress factors for *Sargassum cymosum*, becoming evident in reduced growth rates, an increase in phenolic compounds acting as antioxidants and inhibition of the electron transport rate, despite higher chlorophyll *a* content.

In this study, the chlorophyll *a* content was significantly higher in the glacial run-off area (Fig. 3a); however, this might also be due to other environmental parameters, e.g. reduced PAR availability in run-off plumes (Fig. 1b^59^). Further, we found the antioxidant activity to be significantly lower in the run-off areas (Fig. 3c). This might be due to reduced PAR availability in the run-off plumes, leading to less oxidative stress^60^, or chronic heavy metal exposure resulting in oxidative degradation of antioxidants^45^. We conclude that the sum of environmental factors in the study areas conditioned kelps. The environmental differences and/or altered host conditions might be the reason for changes in the relative abundance of many microbial taxa^36^.

Generally, the macroalgal surface is an attractive substrate for heterotrophic microbial communities, as macroalgae generate oxygen during photosynthesis and further excrete polysaccharides^61^. Burgunter-Delamare et al.^37^ described the microbial community from stressed *S. latissima* to be more similar to each other than to unstressed individuals, which was also confirmed by Marzinelli et al.^36^ for *Ecklonia radiata*. We found that the relative abundances of the kelp-associated microbial taxa strongly depend on the area where the kelps were sampled, with the microbial communities from the run-off areas clustering closer compared to the control area (Fig. 4c). Thereby, the Shannon and Pielou indices (Fig. 4a, b) were lower in the run-off areas compared to the control area, indicating a reduced species richness and evenness. Cundell et al.^62^ stated that the kelp-associated microbial community is not directly related to the free-living microbial community in the water column. We also found distinct differences between the kelp-associated and free-living microbial community, e.g., the absence of Planctomycetes in the water column (Supplementary Fig. 3). Bacteroides, Alphaproteobacteria, and Gammaproteobacteria are considered to be among the most common taxa populating macroalgae^61,63^, which we also confirmed in this study. Rhodobacterales have been described as early colonisers of marine surfaces, with the ability to fix nitrogen^64^. We detected no significant difference in Rhodobacterales between sampling areas. As we used the kelp surface above the meristem for epiphytic microbial analyses, we assume a later stage of microbial succession on the kelp tissue. High abundances of Planctomycetes have been described to be associated with *Laminaria hyperborea* and *Saccharina latissima*^37,63,65^. Planctomycetes have a high number of sulfatases; genes that are described to degrade sulphated polysaccharides^66^. Saprospiraceae were also described to play an important role in metabolising complex carbon resources^67^ and were most abundant in the glacial run-off area.

While this study can neither untangle which (interacting) environmental parameter(s) triggered which kelp response, nor prove a causal link between kelp responses, but only correlations, our data clearly shows high variability in kelp holobiont composition and functioning between run-off systems. This highlights the complexity of kelp responses to Arctic climate change.

The kelp responses have consequences for the entire ecosystem. Being primary producers, kelps serve as food sources for many associated species along Arctic coastlines. As also shown in this study, Pinto et al.^53^ stated that algae accumulate heavy metals at chronic exposure. As a consequence, they found that the algae pass on heavy metals to higher trophic levels. The ingestion of metal contaminated kelps was shown to have negative impacts on the fitness of grazers, such as sea urchins (i.e. growth, fertility, development^68^) and might result in bioaccumulation and biomagnification of heavy metals across the Arctic food web, which could already be shown for *b*Hg^69,70^. As we found the *b*Hg mass fractions in the terrestrial run-off area to be 60–70% higher compared to the other areas, the biomagnification of *b*Hg across the food web might be potentiated when glaciers retreat on land. Macroalgae have long been proposed as biomonitors for the bioavailability of heavy metals, as they are at the basis of the food web and respond strongly to dissolved elements. Commonly used macroalgae for bioindication are e.g. *Ulva*, *Porphyra*, and *Fucus*^71^. Biomonitors offer a direct, time-integrated proxy for the bioavailability of heavy metals in their environment. Our study shows a high potential to use *S. latissima* as a passive biomonitoring organism for heavy metals, being a sedentary and cosmopolitan species that is easy to identify taxonomically^47^.

Altered pigment composition and consequentially photosynthetic output can alter the kelp’s carbon metabolism^72^. Differences in the kelps carbon to nitrogen ratio change the food quality of kelps^73^. Further, altered relative abundances of microbial taxa related to metabolising complex carbohydrates might have consequences for the food web. Brown algae are excreting sulphated polysaccharides as mucus, e.g., fucoidan^74^, which are important for micrograzers and filter-feeders. However, due to its complex structure, it has been described that fucoidan can only be degraded by specialised bacteria and is, therefore highly persistent in the environment^75^. Hence, Buck-Wiese et al.^75^ have proposed fucoidan to contribute an underestimated proportion of kelp related blue carbon. Planctomycetes use fucoidan as a carbon source^65^. Due to their ability to break down complex sugars, they serve as a crucial link in the food web, contributing to the nutritional value of kelps^39^. We found Planctomycetes to have a significantly higher abundance in run-off dominated areas compared to the control area, indicating a higher nutritional value of kelps; however, might alter the ecosystem elemental cycling and eventually decrease kelp carbon sequestration.

Burgunter-Delamare et al.^37^ propose the development of the kelps’ microbiome as a bioindicator with the potential to resolve environmental influences. While kelp individuals from all sampling stations looked healthy in our study, showing no sign of physiological stress the associated microbial community responded to the sum of environmental factors and changes in host conditions. Therefore, we emphasise the necessity to conduct studies assessing kelp holobiont functioning in ecological studies, which has long been suggested (reviewed by^76^).

The high biosorption potential of kelps holds both risks and potentials for high-latitude bio-economical perspectives. Further studies must be conducted to assess their general and local feasibility and ecological consequences. As high-latitude fjords become tolerable for temperate kelp species with ongoing climate change, seaweeds for human consumption have been discussed as a sustainable livelihood possibility^31^. Therefore, their heavy metals biosorption potential has to be considered. Currently, European Union legislation concerning heavy metal maximum levels in kelps is limited, with the exception of France^77^. Kreissig et al.^31^ evaluated the trace metal content of different seaweed groups in Greenland. Even though they found mass fractions of I and Cd to exceed the stricter French regulations, they classified Arctic kelps as a promising food source. Shaughnessy et al.^78^ found Cd and As levels in S. *latissima* to reach critical levels for consumption. While we also found the Cd mass fractions of *S. latissima* to exceed the French regulations of 0.5 µg g_DW_^-1^ in all samples, levels for Pb, Hg, Mn, and Fe were below maximum levels^79^. Thereby, the significant differences in kelp metal content within a small area (Figs. 1a and 2d) are noteworthy and have to be considered regarding the location to implement high-latitude maricultures and evaluating the best suited harvest time. While the biosorption of heavy metals poses a possible risk for food consumption, cultivating *S. latissima* in areas with high heavy metal loads may serve as biomitigation measures, extracting heavy metals from the water column^32^. The harvest of cultivated *S. latissima* in Arctic fjords being dominated by run-off discharge might be a possibility to reduce the risk of biomagnification of biologically harmful element (e.g. *b*Hg) across the Arctic food web. Further, harvesting cultivated kelps in fjords with high run-off and metal load might pose an eco-friendly method for rare earth element mining (algal mining^80^). We found that rare earth element content (*b*La–*b*Lu) responded strongly to sampling station, showing the ratio between biogenic and dissolved element content to be in the magnitude of 10^4^ (Fig. 2c). It has to be considered that this ratio depicts a momentary condition during the sampling time, as dissolved element concentrations in run-off are highly variable^26^. Measuring mean cumulative rare earth element mass fraction, a maximum of 4.3 mg kg_DW_^-1^ was reached. The possibility of phytomining rare earth elements was experimentally tested (e.g., by^81,82^) in several macroalgal species, who highlighted the capabilities of macroalgae as universal biosorbents for rare earth elements.

In conclusion, we found kelps accumulating elements from run-off discharge, correlating with biochemical responses and microbial community changes within a few kilometres area, indicating a high spatial ecological variability. While samples from the glacial and terrestrial run-off areas were more similar to each other compared to the control area, differences between them relate to near-future changes in ecosystem functioning with glaciers retreating (Fig. 6). High contents of harmful elements (e.g. *b*Hg, *b*Cd) in kelps at run-off dominated coastlines are likely to be bioavailable for the Arctic food web. Hence, harmful elements might biomagnify and be potentially passed on to humans. Changes in the kelp-associated microbial community indicated that the holobionts health and functioning is run-off influenced. This might have consequences for the nutritional value of kelps, as well as changes in the ecosystems elemental cycling. We found kelps to be a potential biomonitor for the bioavailability of environmental metals and harmful elements in coastal ecosystems. At the same time, the kelp-associated microbiome could serve as an indicator of the kelp health status and the ecosystem services of the kelp holobiont.

## Methods

### Experimental design

This study was conducted using in-situ samples of the kelp *Saccharina latissima* from Billefjorden. Billefjorden is located on the west coast of Spitsbergen at 78°N. The region between Brucebyen and Kapp Ekholm (Fig. 1A–C) is mostly characterised by relatively clear coastal water. The Nordenskjölbreen glacier (NSB) is terminating into Adolfbukta, discharging glacial run-off (Fig. 1D–F^83^). Petuniabukta (northernmost bay) is characterised by terrestrial run-off gathered by Hørbyedalen, Ragnardalen and Ebbadalen (Fig. 1G–I^84^).

Water column mapping was conducted during mid-day on 30th August 2023. We measured five stations (0–10 m depth; Fig. 1; purple points) within each area, in proximity to, and in between, kelp sampling stations (Fig. 1; yellow points).

Kelp samples were collected between 22 and 30 August 2022. In each area, similar-sized sporophytes of *S. latissima* were sampled at three sampling stations with a plant rake (Plant rake 19.000, acc. to Sigurd Olsen, KC Denmark, Silkeborg, Denmark) on 5 ± 2 m water depth. A schematic overview of the kelp and water sample preparation in the lab is provided in Supplementary Figs. 4 and 5.

### Physical water parameters

We measured spectrally downwelling irradiance (RAMSES-ACC-UV/VIS radiometer, TriOS Optical Sensor, Oldenburg, Germany) from 400 to 700 nm in water depths from 0 to 10 m (alternative calibration). The irradiance of each wavelength was measured. Conversion from mW m^−2^ nm^−1^ to µmol photons m^−2^ s^−1^ and PAR integration were performed after Niedzwiedz & Bischof^24^. The PAR attenuation coefficient (K_d_) was calculated for the PAR after Hanelt et al.^85^ between the surface and 5 m water depth.

CTD profiles were measured with a SWiFT CTDplus Turbidity (Valeport, St Peters Quay, United Kingdom). On each CTD profile, we measured temperature (°C), salinity, and turbidity (NTU). Outliers were removed from the raw data. Trimmed data were smoothed by the median for each full meter.

### Elemental composition

A detailed description of all preparatory work and instrument settings are provided as supporting information. Limits of detection (LODs) and limits of quantification (LOQs) were calculated according to DIN 32645:2008-11 based on method blanks, with LOD defined as 3×standard deviation (SD) and LOQ as 10 × SD.

Water samples for *d*element concentration were taken along with the kelp samples from 5 m depth (Fig. 1a, yellow points), with a trace metal free Niskin bottle (KC Denmark, Silkeborg, Denmark). Four technical replicates of water samples were filtered (DigiFILTER polytetrafluoroethylene (PTFE) membrane, 0.45 μm pore size, PerkinElmer; Waltham, USA) and stabilised using 100 µL concentrated HNO_3_. *d*Elements were measured by using a seaFAST SP2 system (Elemental Scientific; Omaha, USA) coupled online to a triple quadrupole ICP-MS/MS system (Agilent 8900, Agilent Technologies; Tokyo, Japan). Analytes were preconcentrated on two columns filled with Nobias chelate-PA1 resin (HITACHI High-Tech Fielding Corporation; Tokyo, Japan) buffered by 4 mol L^−1^ ammonia acetate buffer (pH = 6.0 ± 0.2) and eluted with 1.5 mol L^−1^ HNO_3_. To correct for instrumental drift, a 1 µg L^−1^ Niob (Nb) solution was used as an internal standard. As certified reference material AQUA-1, SLEW-4 and NASS-7 (National Research Council Canada; Ottawa, Canada) were used for method validation (recovery rates; LOD; LOQ: Supplementary Table 1).

Kelp material (~ 10 cm wide stripe above meristem) for *b*element analyses, was rinsed with ultrapure water and freeze-dried, before powdering and homogenising with a ball mill (Agate; Planeten Kugelmühle PM400, Retsch; Düsseldorf, Germany). Of each sample, 100 mg of three technical replicates were weighed into 55 mL TFM (modified PTFE) digestion vessels. For digestion, 0.1 mL HBF_4_, 5 mL HNO_3_, 2 mL HCl and 1 mL H_2_O_2_ were added (adapted from^86^) and TFM vessels were placed in a closed-vessel microwave-assisted digestion system (Mars 6, CEM Corporation; Matthews, USA). The microwave was set to reach a maximum of 200 °C after a suitable and efficient temperature ramping (Supplementary Table 2). The sample digests were quantitatively transferred into 50 mL DigiTubes and diluted to 50 mL with ultrapure water. Along with each batch, two blank digestion vessels containing only reagents were processed to monitor procedural contaminations and carry-over effects. For method validation, CRM NIST-3232 (Kelp powder *Thallus laminariae*, National Institute of Standards and Technology, Gaithersburg, USA) was digested under the same conditions (recovery rates; LOD; LOQ: Supplementary Table 3). The digested samples were measured with a triple quadrupole ICP-MS/MS system (Agilent 8800, Agilent Technologies; Tokyo, Japan) coupled to an ESI SC-4DX FAST autosampler (Elemental Scientific; Omaha, USA). The recovery of all certified elements was between 80 and 120%. The standard deviation of all non-certified elements between measurements was 2–90% of the mean (Supplementary Table 3). Element mass fraction in kelps was calculated as µg g_DW_^–1^^87^.

To estimate the transfer potential of rare earth elements from the water column to kelps^88^, we calculated the ratio of biogenic rare earth element mass fraction (µg g_DW_^–1^) to dissolved element concentration in 5 m water depth (µg mL^−1^)^89^.

### Biochemical composition

Algal pigment content responds to PAR availability and cellular energy requirements^90^. Pigment composition was determined after Koch et al.^91^. 30 mg silica-dried, powdered, meristematic material (*N* = 6–9) were analysed and dark extracted in 1 mL 90% Acetone for 24 h at 4 °C. The filtered supernatant was analysed by a High-Performance Liquid Choromatography (HPLC; LaChromElite^®^ system, L-2200 autosampler (chilled), DA-detector L-2450; VWR-Hitachi International GmbH, Darmstadt, Germany). The pigments were separated after a gradient according to Wright et al.^92^, by a Spherisorb^®^ ODS-2 column (250 × 4.6 mm, 5 μm; Waters, Milford, MA, USA). Respective standards were used to identify and quantify pigment peaks (DHI Lab Products, Hørsholm, Denmark). The accessory pigments were calculated as the sum of chlorophyll c, fucoxanthin and β-carotene. The ratio of accessory pigments to chlorophyll *a* was calculated. Pigment contents were calculated in µg g_DW_^−1^. The de-epoxidation state of xanthophyll cycle pigments (DPS) was calculated after Colombo-Pallotta et al.^93^.

Antioxidants serve as a mechanism of protection against environmental stressors^60^. Antioxidant activity was determined after Re et al.^94^, following the ABTS^·+^ (2,2’-azino-bis-3-ethylbenzthiazoline-6-sulphonic acid, 7 mM in biDest H_2_O) assay. One aliquot of 50 mg silica-dried, powdered, meristematic material (*N* = 6–9) was dark extracted in 1 mL 70% Ethanol for 4 h at 47 °C. 10 µL of the supernatant were mixed with 1 mL ABTS^·+^-working-standard (absorption range: 0.740 ± 0.01;734 nm). The absorption (734 nm) was measured after 6 min incubation. Antioxidant activity was calculated as Trolox-equivalents (TE) by calibrating the ABTS^·+^-working-solution with a Trolox dilution series (6-hydroxy-2,5,7,8-tetramethychroman-2-carboxylic acid; 2.5 mM in 70% Ethanol).

### Microbial community

An area of 10 × 10 cm above the meristem was swabbed with a sterile cotton swab and immediately frozen at -80°C until analysis. Bacterial DNA was extracted using the QIAamp DNA Mini Kit (QIAGEN, Hilden, Germany) following the manufacturer’s instructions. Qubit (ThermoFisher Scientific, Darmstadt, Germany) was used to detect the concentrations of extracted DNA. For microbial community composition analysis, the V4–V5 regions (515F: 5’-GTGCCAGCMGCCGCGGTAA-3’ and 907R: 5’-CCGTCAATTCMTTTRAGTTT-3’) of the bacterial 16 S rRNA gene from DNA extracts were amplified using the PCR procedure^95,96^. Quantified amplicons were sequenced using the Illumina Nova platform (Shanghai Hanyu Biotech lab, Shanghai, China), generating 250 bp paired-end reads. Raw reads were quality filtered using Trimmomatic (v.39) with standard parameters^97^. Amplicon Sequence Variant (ASV) were clustered based on high-quality sequences with a 100% similarity and then taxonomically classified based on the Greengenes database (v13.8^98^). To ensure comparability of subsequent analysis, these ASVs were rarified to 49,918 sequences per sample for α diversity analyses.

### Statistical analysis

All statistical analyses were run in RStudio (V 2023.12.1 using R-4.3.2-win^99^). Data were evaluated and plotted within “tidyverse”^100^. Section plots and maps were created with “PlotSvalbard”^101^ All reported values were rounded to significant digits.

Outliers were removed from the raw data if classified as extreme (deviation > 3 x interquartile range; function: identify_outliers; package: rstatix^102^) to account for possible sampling and processing errors. A linear model was fit on each response parameter (function: lm; package: stats^99^). Sampling area and station were modelled as single fixed effects, to analyse spatial differences of kelp responses. The normality (Shapiro-Wilk test, *P* > 0.05) and homoscedasticity (Levene’s test, *P* > 0.05) of model residuals were tested in addition to visual assessment via histograms. As the data met requirements, analysis of variance was performed on the model by using the “anova” function (type I sums of squares). Pairwise performance (function: emmeans; package: emmeans^103^) was used to calculate the degrees of freedom, with Tukey adjustment of the p-value. Pearson correlation analyses were calculated (function: cor.test; package: stats^99^). The correlogram was plotted using the ggcorr-function (package: GGally^104^).

Using non-metric multidimensional scaling (nMDS), kelp population structures between areas were tested, based on Bray-Curtis dissimilarities. Variables were fit onto unconstrained ordinations (function: envfit; package: vegan^105^) to explore relationships between kelp responses and environmental drivers.

## Electronic supplementary material

Below is the link to the electronic supplementary material.

Supplementary Material 1

## Data Availability

All data supporting this study are openly available. Water parameters (temperature, salinity, turbidity, available PAR, element concentration, microbial community): https://doi.pangaea.de/10.1594/PANGAEA.968625. Kelp responses (pigments, antioxidant activity, elemental mass fraction): https://doi.pangaea.de/10.1594/PANGAEA.968627. Elemental content of certified reference material is provided as Supporting information. The microbial sequences obtained in this study have been deposited in the NCBI SRA database under the ID number: PRJNA1097779.
