# Supplementary material for: Run-off impacts on Arctic kelp holobionts have strong implications on ecosystem functioning and bioeconomy
Source: Sci Rep. 2024 Dec 16;14:30506. doi: 10.1038/s41598-024-82287-w (PMC11649688; doi:10.1038/s41598-024-82287-w)
Supplement: Supplementary file 1 — Supplementary Material 1 [file 41598_2024_82287_MOESM1_ESM.docx]

Supplementary material to:

**Run-off impacts on Arctic kelp holobionts have strong implications on ecosystem functioning and bioeconomy**

**Short title**: Run-off impacts on Arctic kelp holobionts

Authors: Sarina Niedzwiedz^1*^, Claudia Schmidt^2,3^, Yunlan Yang^4^, Bertille Burgunter-Delamare^5^, Sebastian Andersen^6^, Lars Hildebrandt^7^, Daniel Pröfrock^7^, Helmuth Thomas^2,3^, Rui Zhang^4^, Børge Damsgård^6^, Kai Bischof^1^

***corresponding author**: sarina@uni-bremen.de

Affiliations: ^1^Marine Botany, Faculty of Biology and Chemistry & MARUM, University of Bremen, 28359 Bremen, Germany.

^2^Institute for Chemistry and Biology of the Marine Environment (ICBM), University of Oldenburg, 26111 Oldenburg, Germany.

^3^Helmholtz-Zentrum Hereon, Institute of Carbon Cycles, Department of Marine Carbon Cycles, 21502 Geesthacht, Germany.

^4^Archaeal Biology Center, Synthetic Biology Research Center, Shenzhen Key Laboratory of Marine Microbiome Engineering, Key Laboratory of Marine Microbiome Engineering of Guangdong Higher Education Institutes, Institute for Advanced Study, Shenzhen University,518052 Shenzhen, China.

^5^Matthias Schleiden Institute of Genetics, Bioinformatics and Molecular Botany, Friedrich Schiller University Jena, 07743 Jena, Germany.

^6^The University Centre of Svalbard (UNIS), 9171 Longyearbyen, Norway.

^7^Helmholtz-Zentrum Hereon, Institute of Coastal Environmental Chemistry, Department Inorganic Environmental Chemistry, 21502 Geesthacht, Germany.

Supplementary Methods

Preparatory laboratory work

Preparatory laboratory work was performed in a class 10000 or 1000 clean room. Type I reagent-grade water (18.2 MΩ cm, ultrapure water) was obtained from a Milli-Q Integral water purification system equipped with a Q-Pod Element and a 100 nm endfilter (Merck; Darmstadt, Germany). P.a. grade concentrated nitric acid (HNO3; ROTIPURAN®, w = 65 %, Carl Roth GmbH + Co. KG; Karlsruhe, Germany) and hydrochloric acid (HCl; ROTIPURAN®, w = 37 %, Carl Roth GmbH + Co. KG; Karlsruhe, Germany) were purified by double sub-boiling using a perfluoroalkoxy alkane (PFA) acid purification systems (Savillex; Eden Prairie, USA) operated under clean room conditions. Glacial acetic acid (C₂H₄O₂; Optima™, Fisher Scientific GmbH; Schwerte, Germany), Ammonia solution (NH3; Optima™, w = 20–22 %, Fisher Scientific; Schwerte, Germany), hydrogen peroxide solution (H2O2; ROTIPURAN® Ultra, w = 31 %, Carl Roth GmbH + Co. KG; Karlsruhe, Germany) and tetrafluoroboric acid (HBF_4_; ultrapure, w = 38 %, Chem-lab NV; Zedelgem, Belgium) were used without further purification. For quantification of analytes, external calibrations were performed using single element standards (Carl Roth GmbH; Karlsruhe, Germany or Sigma-Aldrich; Missouri, USA) and custom-made multi-element standards (all traceable to NIST standards) of different compositions (Inorganic Ventures; Christiansburg, USA) to cover the targeted analyte concentration ranges. All plastic consumables were pre-cleaned in solutions of HNO_3_ (w = 1–2 %) for a minimum of one week and rinsed with ultrapure water prior to use. Microwave digestion vessels were cleaned (2×) in a steam cleaner at 90°C for 8 h (65 % HNO_3_; Easy Trace Cleaner Evolution II, ANALAB, Paris, France).

Analysis of dissolved elements in water samples

Water samples (elemental composition, microbial community) were taken along with the kelp samples (**Figure 1**; yellow points), with a trace metal free Niskin bottle (KC Denmark, Silkeborg, Denmark). In total, 18 fjord water samples were collected using a 1.7 L trace-metal free Niskin water sampler (KC Denmark; Silkeborg, Denmark), filled into 0.5 L high-density polyethylene (HDPE) bottles and kept frozen (-20°C) until further processing. Prior to multi-element analysis, water samples were filtered through DigiFILTERs™ (polytetrafluoroethylene (PTFE) membrane, 0.45 μm pore size, PerkinElmer; Waltham, USA) and collected in 50 mL DigiTUBE®s (PerkinElmer; Waltham, USA). After filtration, water samples were stabilized using 100 µL purified concentrated HNO3 and stored in the dark at 4°C until analysis.

Elements in seawater were measured by using a seaFAST SP2 system (Elemental Scientific; Omaha, USA) coupled online to a triple quadrupole ICP-MS/MS system (Agilent 8900, Agilent Technologies; Tokyo, Japan). Analytes were preconcentrated on two columns filled with Nobias chelate-PA1 resin (HITACHI High-Tech Fielding Corporation; Tokyo, Japan) buffered by 4 mol L^-1^ ammonia acetate buffer (pH = 6.0±0.2) and eluted with 1.5 mol L^-1^ HNO_3_. To correct for instrumental drift, a 1 µg L^-1^ Niob (Nb) solution was used as an internal standard.

The ICP-MS instrument was optimized daily using a tuning solution containing Li, Co, Y, Ce and Tl to maintain a reliable day-to-day performance. The system was operated in He/H_2_ mode and equipped with a x-lense. The certified reference materials (CRMs) AQUA-1 for drinking water, SLEW-4 for estuarine water and NASS-7 for open ocean seawater (all provided by National Research Council Canada; Ottawa, Canada) were used for method validation. Recovery rates (between 80 % and 150 %) are provided in **Table S1**.

Analysis of elements in kelp samples

After sampling, the kelp material (approx. 10 cm wide stripe above meristem; cut with ceramic knife) was rinsed with ultrapure water and freeze-dried for 72 h. It was powdered and homogenized using a ball mill (Agate; Planeten Kugelmühle PM400, Retsch; Düsseldorf, Germany). Of each sample, three aliquots of 100 mg were weighed into 55 mL TFM (modified PTFE) digestion vessels (MARS6, CEM Corporation; Matthews, USA). For digestion, 0.1 mL HBF_4_, 5 mL HNO_3_, 2 mL HCl and 1 mL H_2_O_2_ were added (adapted from 85). Afterwards, the TFM vessels were placed in a closed-vessel microwave-assisted digestion system (Mars 6, CEM Corporation; Matthews, USA). The microwave was set to reach a maximum of 200°C after a suitable and efficient temperature ramping (**Table S2**).

The sample digests were transferred quantitatively into 50 mL DigiTUBE®s (PerkinElmer; Waltham, USA) and diluted to 50 mL with ultrapure water to reduce acid concentration. Along with each batch, two blank digestion vessels containing only reagents were processed to monitor procedural contaminations and carry-over effects. Furthermore, two vessels containing the CRM NIST-3232 (Kelp powder *Thallus laminariae*, National Institute of Standards and Technology; Gaithersburg, USA) were digested for method validation. Recovery rates are contained in **Table S3** (between 80–121 %). In addition to the certified elements numerous non-certified elements were quantified.

The digested samples were measured by means of a triple quadrupole ICP-MS/MS system (Agilent 8800, Agilent Technologies; Tokyo, Japan) coupled to an ESI SC-4DX FAST autosampler (Elemental Scientific; Omaha, USA). To correct for instrumental drift, an Iridium (Ir)/Rhodium (Rh) solution (10 µg L^−1^) was used as an internal standard. The ICP-MS instrument was optimized daily using a tuning solution containing Li, Co, Y, Ce and Tl to maintain a reliable day-to-day performance. The system was operated in 5 different gas modes and equipped with a x-lense. In addition to the CRM, an in-house reference multi-element solution comprising different single-element standards and custom-made multi-element standards was used for validation with respect to elements not certified for the CRM.


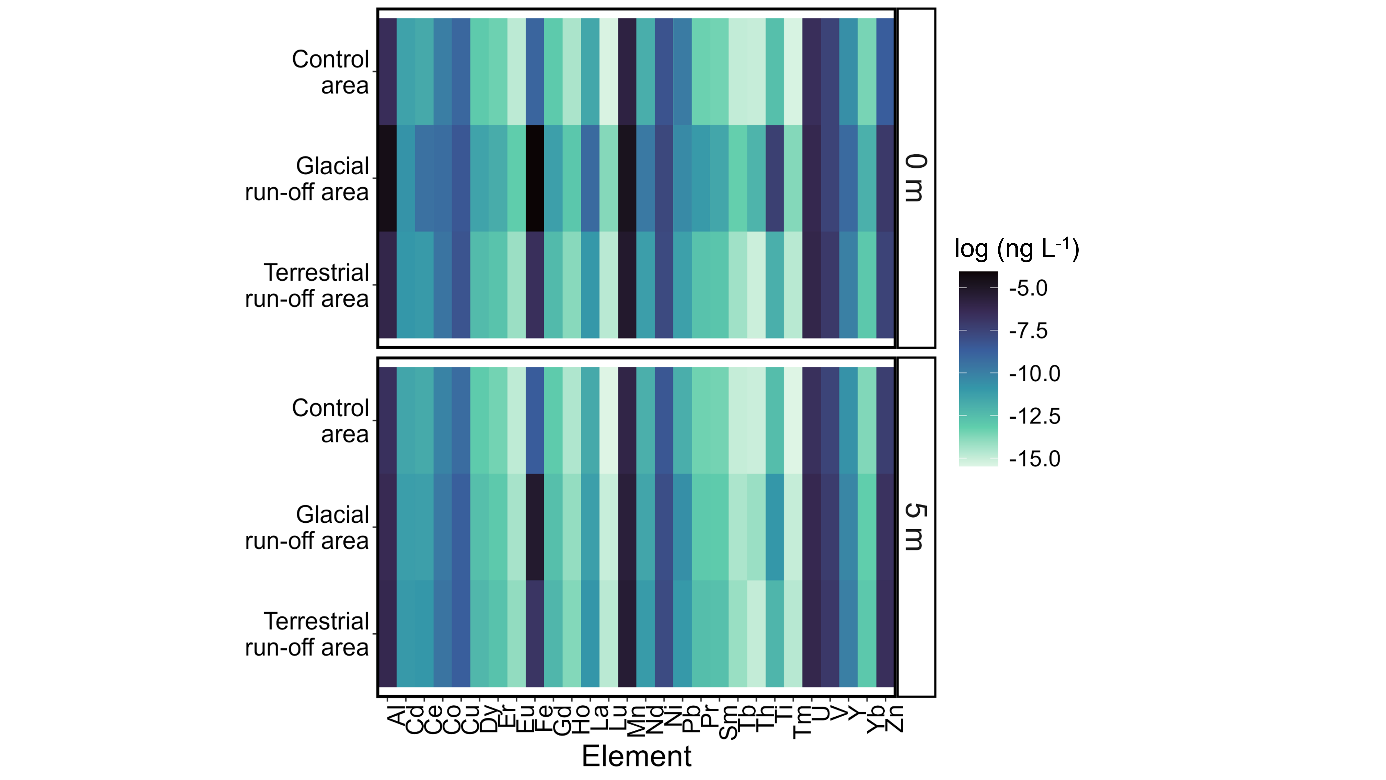


Supplementary Figure 1. Concentration (ng L^-1^) of dissolved elements on 0 m and 5 m depth in the control, glacial run-off and terrestrial run-off area. Concentration is shown as log(ng L^-1^) to highlight low concentrations.


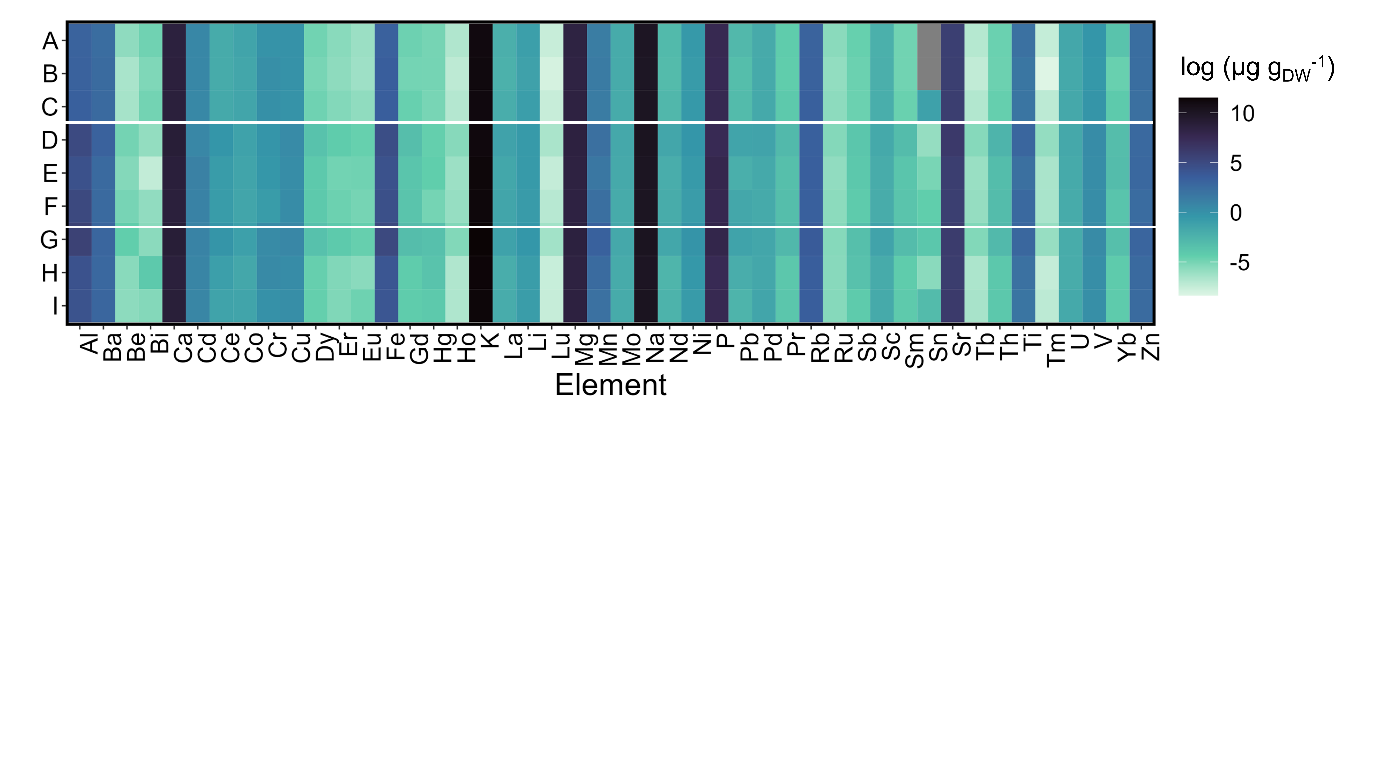


**Supplementary Figure** **2.** Mass fractions (µg g_DW_^-1^) of kelp elements depending on sampling station. A–C: Control area. E–F: Glacial run-off- G–I: Terrestrial run-off. A) Heatmap of all determined elements (alphabetically ordered) in kelps. Mass fraction is shown as log(µg g_DW_^-1^) to highlight low mass fractions.


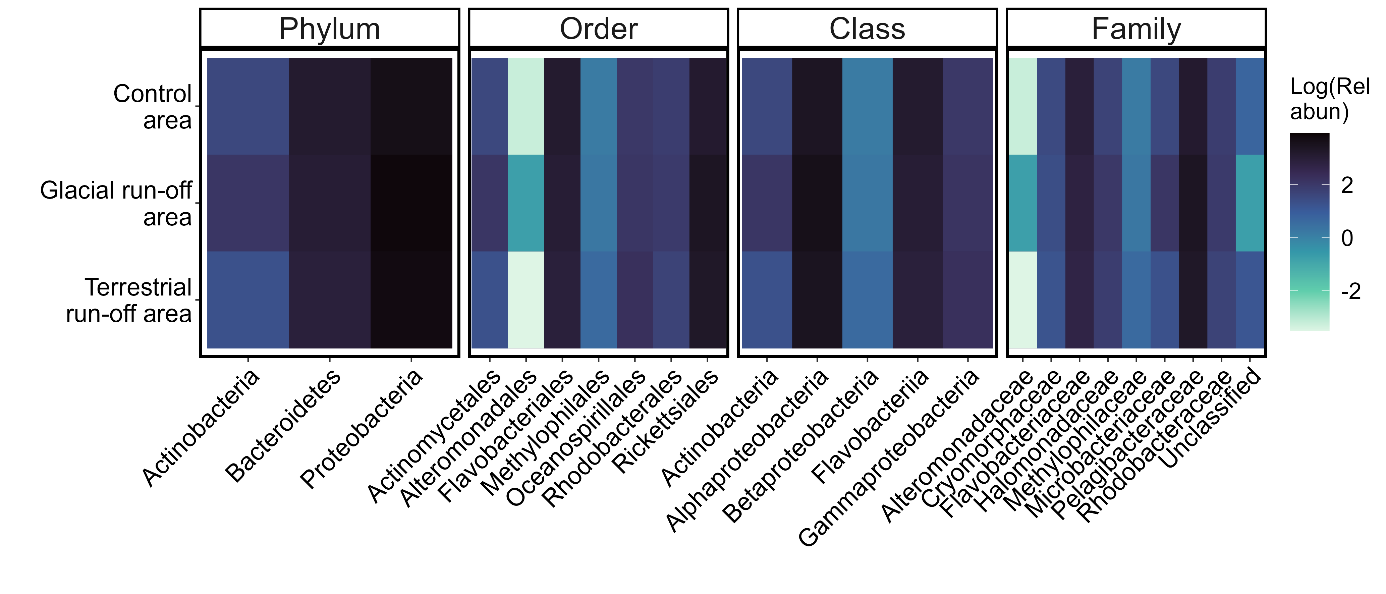


**Supplementary Figure 3.** Heatmap showing the mean relative abundance of free-living microbial community taxa (≥ 1 %) in each sampling area on 5 m water depth. Relative abundance is shown as Log(Rel. abun.) to highlight low abundances.


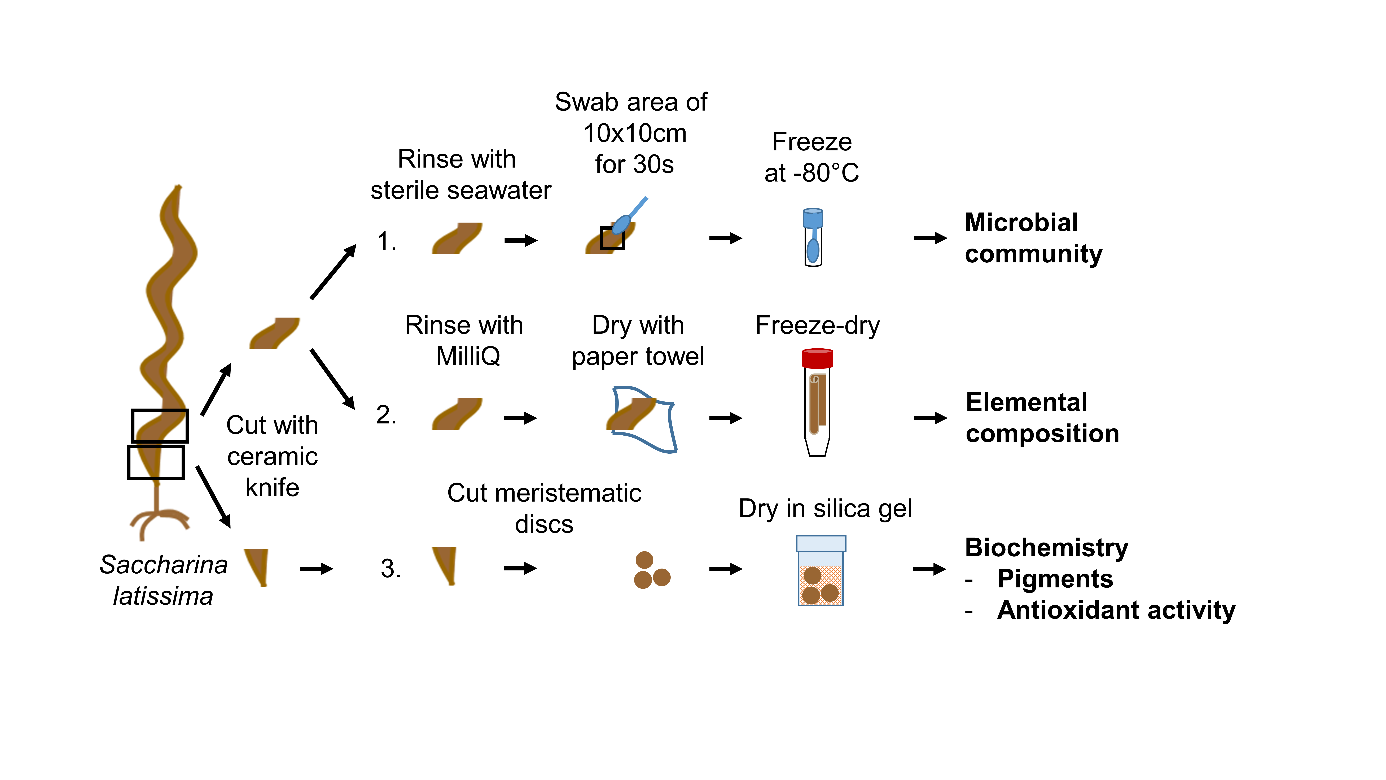


Supplementary Figure 4. Schematic preparation of kelp samples. 1. Microbial community: Swab area of 10×10 cm above meristem with a sterile cotton swab for 30 s and freeze samples on -80 °C until analysis. 2. Elemental composition: Rinse area above meristem with type I reagent grade water and freeze-dry. 3. Biochemistry: Dry meristematic discs in silica gel for pigment analysis and antioxidant activity.


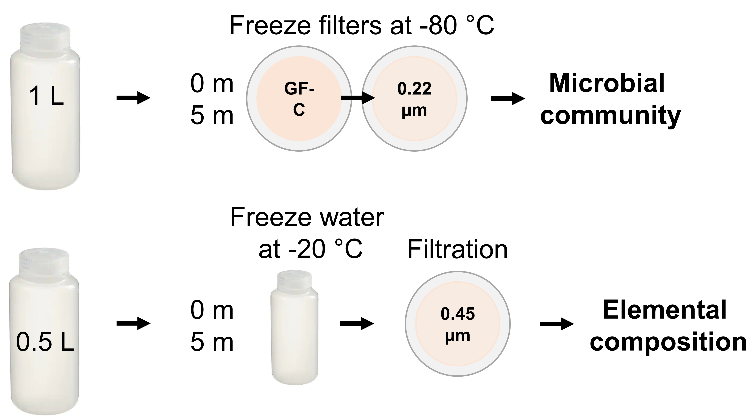


Supplementary Figure 5. Schematic procession of water samples from surface and 5 m depth. Microbial community: Prefilter on GF-C before sterile filtration on 0.22 µm sterile filter. Elemental composition: Freeze water on -20°C and filter on 0.45 µm filter before analyses.

**Supplementary Table 1**. Recovery (%) of all certified and non-certified elements of CRM AQUA-1, SLEW-4, NASS-7 (*N*=4). Limits of detection (LOD; ng L^-1^) and limits of quantification (LOQ; ng L^-1^) were calculated according to DIN 32645:2008-11 based on 14 method blanks (*N*=14), with LOD defined as 3 × standard deviation (SD) and LOQ as 10 × SD of the blank.

| **Element** | **Recovery (%)** | | | **Concentration (ng L^-1^)** | |
| --- | --- | --- | --- | --- | --- |
|  | **AQUA-1** | **SLEW-4** | **NASS-7** | **LOD** | **LOQ** |
| **Al** | 85.42 | - | 163.15 | 30.86 | 43.91 |
| **Ti** | 83.26 | - | 136.72 | 1.62 | 2.39 |
| **V** | 110.12 | 103.24 | 94.37 | 0.34 | 0.80 |
| **Mn** | 107.94 | 96.98 | 98.35 | 4.40 | 13.77 |
| **Fe** | 104.69 | 96.83 | 99.90 | 71.18 | 79.66 |
| **Co** | 94.74 | 103.38 | 97.46 | 0.05 | 0.12 |
| **Ni** | 108.09 | 101.59 | 95.41 | 0.79 | 1.21 |
| **Cu** | 105.75 | 103.67 | 95.33 | 2.81 | 8.39 |
| **Zn** | 108.57 | 104.43 | 102.84 | 12.00 | 32.19 |
| **Y** | 116.89 | - | 108.50 | 0.00 | 0.01 |
| **Cd** | 148.43 | 104.84 | 94.73 | 0.06 | 0.11 |
| **La** | 111.04 | - | 105.08 | 0.01 | 0.02 |
| **Ce** | 110.45 | - | 105.59 | 0.02 | 0.05 |
| **Pr** | 111.49 | - | 105.87 | 0.00 | 0.00 |
| **Nd** | 104.53 | - | 100.45 | 0.01 | 0.02 |
| **Sm** | 103.25 | - | 98.62 | 0.00 | 0.00 |
| **Eu** | 110.34 | - | 104.22 | 0.01 | 0.02 |
| **Gd** | 142.35 | - | 108.24 | 0.01 | 0.02 |
| **Tb** | 135.24 | - | 115.18 | 0.00 | 0.01 |
| **Dy** | 111.11 | - | 102.73 | 0.00 | 0.00 |
| **Ho** | 112.63 | - | 106.29 | 0.00 | 0.01 |
| **Er** | 106.80 | - | 101.24 | 0.00 | 0.01 |
| **Tm** | 111.67 | - | 110.32 | 0.00 | 0.01 |
| **Yb** | 105.84 | - | 100.57 | 0.00 | 0.01 |
| **Lu** | 120.95 | - | 114.46 | 0.00 | 0.01 |
| **Pb** | 110.82 | 110.76 | 122.38 | 0.33 | 0.86 |
| **Th** | 38.87 | - | 69.45 | 0.09 | 0.27 |
| **U** | 113.15 | 89.29 | 79.99 | 0.04 | 0.12 |

Supplementary Table 2. Microwave temperature ramping for kelp digestion

| **Stage** | **Ramp (min)** | **Hold (min)** | **Temperature (°C)** | **Power (W)** |
| --- | --- | --- | --- | --- |
| 1 | 05:00 | 10:00 | 50 | 400 |
| 2 | 10:00 | 30:00 | 20 | 400 |
| 3 | 30:00 | 30:00 | 50 | 400 |
| 4 | 30:00 | 59:59 | 200 | 1600 |
| 5 | 10:00 | 30:00 | 100 | 800 |

Supplementary Table 3. Mass fractions (mg g_DW_^-1^) of all certified and non-certified elements of CRM Kelp powder *Thallus laminariae*, SKU: 3232, NIST, USA (*N*=19). Limits of detection (LODs) and limits of quantification (LOQs) were calculated according to DIN 32645:2008-11 based on 14 method blanks (*N*=14), with LOD defined as 3 × standard deviation (SD) and LOQ as 10 × SD of the blank.

| **Elements** | **Mean**  **(µg g_DW_^-1^)** | **±SD** | **Recovery CRM 3232 (%)** | **LOD**  **(µg g_DW_^-1^)** | **LOQ**  **(µg g_DW_^-1^)** |
| --- | --- | --- | --- | --- | --- |
| **Certified elements** | | | | | |
| **Ca** | 12 473 000 | 1 084 001 | 102 | 9.98 | 33.3 |
| **Cd** | 390 | 60 | 93 | 0.0155 | 0.0517 |
| **Cr** | 6000 | 970 | 101 | 1.07 | 3.57 |
| **Cu** | 3 100 | 580 | 80 | 0.547 | 1.82 |
| **Fe** | 685 000 | 36 600 | 102 | 7.26 | 24.2 |
| **Hg** | 110 | 12 | 93 | 0.115 | 0.382 |
| **K** | 77 000 000 | 8 126 000 | 101 | 1110 | 3690 |
| **Mg** | 5 915 000 | 498 100 | 97 | 3.64 | 12.1 |
| **Mn** | 29 660 | 3 780 | 121 | 5.53 | 18.4 |
| **Mo** | 230 | 23 | 94 | 0.0545 | 0.182 |
| **Na** | 15 500 000 | 1 260 000 | 95 | 13.7 | 45.8 |
| **Pb** | 1 030 | 150 | 99 | 0.129 | 0.429 |
| **Zn** | 26 940 | 1 980 | 98 | 1.66 | 5.52 |
| **Non-certified elements** | | | **Deviation SD from mean (%)** |  | |
| **Al** | 1 070 000 | 20 600 | 1.9 | 9.98 | 33.3 |
| **Ba** | 72 200 | 6 260 | 8.7 | 0.0495 | 0.165 |
| **Be** | 35 | 7 | 20.0 | 0.000434 | 0.00145 |
| **Bi** | 27 | 9 | 33.3 | 0.00782 | 0.0261 |
| **Ce** | 1 550 | 210 | 13.5 | 0.0348 | 0.116 |
| **Co** | 305 | 33 | 10.8 | 0 | 0 |
| **Dy** | 75 | 11 | 14.7 | 0.00162 | 0.00538 |
| **Er** | 42 | 7 | 16.7 | 0.0017 | 0.00566 |
| **Eu** | 25 | 3 | 12.0 | 0.00596 | 0.0199 |
| **Gd** | 80 | 13 | 16.3 | 0.000843 | 0.00281 |
| **Ho** | 13 | 2 | 15.4 | 0.000267 | 0.00089 |
| **La** | 660 | 90 | 13.6 | 0.0163 | 0.0544 |
| **Li** | 1 150 | 220 | 19.1 | 0.505 | 1.68 |
| **Lu** | 7 | 1.4 | 20.0 | 0.000485 | 0.00162 |
| **Nd** | 480 | 80 | 16.7 | 0.0125 | 0.0417 |
| **Ni** | 2 700 | 320 | 11.9 | 0.0323 | 0.108 |
| **P** | 4 200 000 | 545 000 | 13.0 | 20 | 66.8 |
| **Pd** | 400 | 50 | 12.5 | 0 | 0 |
| **Pr** | 130 | 20 | 15.4 | 0.000728 | 0.00243 |
| **Rb** | 25 400 | 4 490 | 17.7 | 0.0175 | 0.0584 |
| **Ru** | 3 | 1.4 | 46.7 | 0.0178 | 0.0593 |
| **Sb** | 60 | 9 | 15.0 | 0.000306 | 0.00102 |
| **Sc** | 440 | 150 | 34.1 | 0.00295 | 0.00985 |
| **Sm** | 95 | 17 | 17.9 | 0 | 0 |
| **Sn** | 90 | 24 | 26.7 | 0 | 0 |
| **Sr** | 820 200 | 122 700 | 15.0 | 0.0859 | 0.286 |
| **Tb** | 11 | 1.2 | 10.9 | 0 | 0 |
| **Th** | 170 | 21 | 12.4 | 0.00332 | 0.0111 |
| **Ti** | 43 300 | 4 350 | 10.0 | 27.5 | 91.8 |
| **Tm** | 8 | 4 | 50.0 | 0.000348 | 0.00116 |
| **U** | 230 | 25 | 10.9 | 0.00312 | 0.0104 |
| **V** | 4 560 | 610 | 13.4 | 0.468 | 1.56 |
| **Yb** | 95 | 85 | 89.5 | 0.0136 | 0.0452 |

**References:**

DIN e.V. “Chemical analysis – Decision limit, detection limit and determination limit under repeatability conditions: Terms, methods, evaluation”. DIN 32645:2008-11 (2008). [no author]
